# Association between red cell distribution width-to-albumin ratio and acute kidney injury in acute pancreatitis: A retrospective cohort study from the MIMIC-IV database

**DOI:** 10.1097/MD.0000000000044810

**Published:** 2025-10-03

**Authors:** Chan-Juan Zhou, Si-Ming Lin, Jian-Tao Zheng

**Affiliations:** a Department of General Practice, Quanzhou First Hospital Affiliated to Fujian Medical University, Quanzhou, China; b Department of Emergency Medicine, The First Affiliated Hospital to Fujian Medical University, Fuzhou, China; c Department of Emergency Medicine, Quanzhou First Hospital Affiliated to Fujian Medical University, Quanzhou, China.

**Keywords:** acute kidney injury, acute pancreatitis, albumin, MIMIC-IV database, red cell distribution width, red cell distribution width to albumin ratio

## Abstract

This study aimed to evaluate the association between the red cell distribution width-to-albumin ratio (RAR) and the early onset of Acute Kidney Injury (AKI) in patients diagnosed with acute pancreatitis (AP). A retrospective cohort study was conducted using data from the Medical Information Mart for Intensive Care IV database, focusing on the first 24 hours following admission to the intensive care unit (ICU). The primary outcome was the incidence of AKI within 7 days of ICU admission, as defined by the Kidney Disease: Improving Global Outcomes criteria. Logistic regression models were employed to assess the association, with subgroup analyses considering variables such as age, sex, renal disease, diabetes, sepsis, use of mechanical ventilation, and sequential organ failure assessment scores. The analysis included 599 patients. Each unit increase in RAR was linearly associated with a 62% greater likelihood of AKI within 7 days of ICU admission (odds ratio = 1.62, 95% confidence interval [CI]: 1.34–1.96, *P* < .010). When RAR was categorized into tertiles, a graded increase in AKI risk was observed. Patients in the middle tertile had a 2.13-fold higher risk of AKI (95% CI: 1.3–3.5, *P* = .003), while those in the highest tertile demonstrated a 3.64-fold increased risk (95% CI: 2.58–8.33, *P* < .001) compared to the lowest tertile. The association persisted across all examined subgroups, highlighting the robustness of RAR as a predictive factor for AKI in patients with AP. RAR demonstrated a significant association with the early onset of AKI in patients with AP admitted to the ICU. These findings highlight the potential of RAR as a reliable predictor for AKI in this patient population.

## 1. Introduction

Acute pancreatitis (AP) is among the most common causes of acute abdomen. The annual incidence of AP is approximately 34 cases per 100,000 individuals in the general population.^[1]^ In severe cases, AP may progress to multi-organ dysfunction, with acute kidney injury (AKI) being a frequent and sever complication. Patients with AP who develop AKI experience significantly higher mortality rates compared to those without AKI.^[2]^ Therefore, early identification of AKI in patients with AP is essential for the timely implementation of targeted interventions.

The red cell distribution width-to-albumin ratio (RAR, %/g/dL) has emerged as a potential inflammatory biomarker. Defined as the ratio of red cell distribution width (RDW) to serum albumin levels, RAR has been associated with adverse outcomes in various inflammatory diseases in prior studies.^[3–5]^ However, the relationship between RAR and the risk of AKI in patients with AP remains unclear.

This study aims to evaluate the association between RAR and the risk of AKI in patients diagnosed with AP, aiming to provide insights into the utility of RAR as a predictive biomarker for AKI in this context.

## 2. Methods

### 2.1. Data source

This retrospective cohort study used data from the Medical Information Mart for Intensive Care IV (MIMIC-IV) database, version 2.2. The MIMIC-IV database contains detailed hospitalization records from patients admitted to a tertiary academic medical center in Boston, MA, USA, between 2008 and 2019. The MIMIC-IV database is a comprehensive repository of healthcare information, including 431,231 hospital admissions and 73,181 intensive care unit (ICU) stays from 299,712 unique patients. It contains a wide range of clinical data, such as laboratory test results, medication administration records, and documentation of vital signs.^[6]^ This study was conducted in accordance with the Declaration of Helsinki. The MIMIC-IV database has been approved by the Massachusetts Institute of Technology and the Beth Israel Deaconess Medical Center, and publicly accessible copies of these data can be found in the MIMIC database. Therefore, this study does not require separate ethical approval or informed consent.

Access to MIMIC-IV does not require patient consent. Researchers who have completed the Collaborative Institutional Training Initiative program are granted access to the data. All authors of this study have fulfilled this requirement, with the following certification numbers: Jiantao Zheng (56456740), Chanjuan Zhou (56513243), and Siming Lin (49037863). Given the public nature of the MIMIC-IV database and the retrospective design of this study, the Quanzhou First Hospital Affiliated with Fujian Medical University exempted this research from ethical approval and patient consent requirements.

This study adheres to the Strengthening the Reporting of Observational Studies in Epidemiology guidelines, ensuring transparency and rigor in the reporting process.^[7]^

### 2.2. Study participant selection criteria

**Figure 1.**
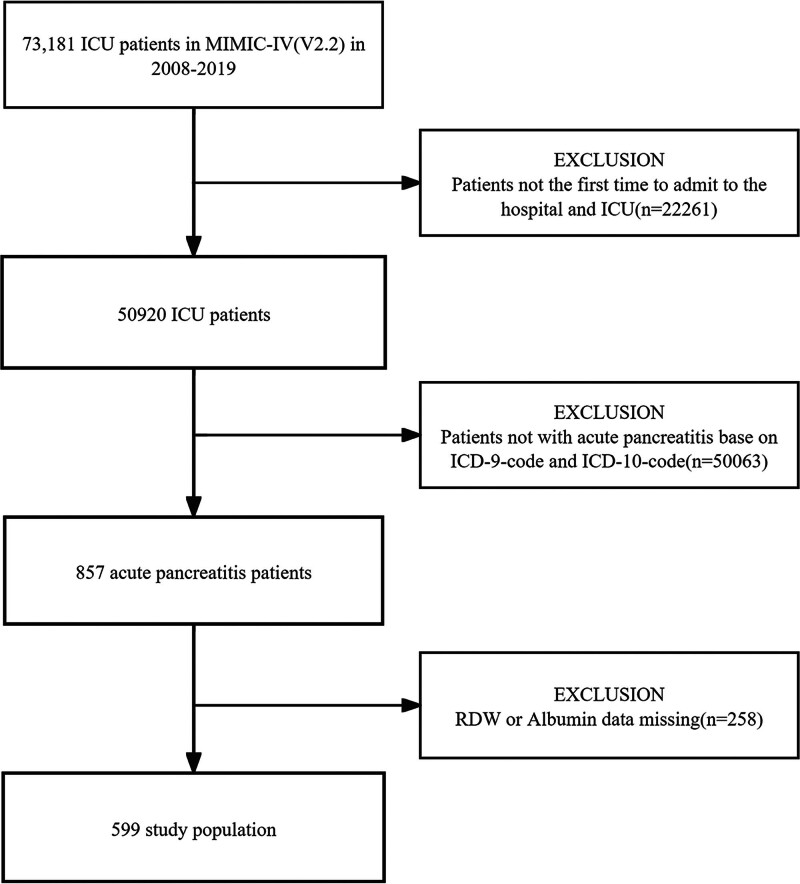
Flowchart of study patients. ICU = intensive care database, MIMIC = medical information mart for intensive care, RAR = red blood cell distribution width-to-albumin ratio.

*Inclusion criteria*: adult patients aged 18 years or older with a primary diagnosis of AP at the time of their initial ICU admission were included in the study. Diagnoses were identified using International Classification of Diseases, Ninth and Tenth Revisions (ICD-9 and ICD-10) coding systems. To maintain analytical integrity, only the first ICU admission was considered for patients with multiple ICU encounters. *Exclusion criteria*: participants with incomplete medical records, particularly those missing key data on albumin and RDW values, were excluded from the analysis. Details are illustrated in Figure 1.

### 2.3. Variable extraction

A comprehensive set of variables was extracted for this study, encompassing demographic characteristics and clinical data. Demographic variables included (such as age, sex, and race). Clinical data recorded upon ICU admission included vital signs, such as mean arterial pressure and respiratory rate, and laboratory parameters, including white blood cell count, platelet count, RDW, serum albumin, anion gap, serum creatinine (SCr), blood urea nitrogen, glucose, total bilirubin, electrolytes (calcium, chloride, and potassium), and activated partial thromboplastin time (APTT).

Comorbidities were extensively cataloged, including congestive heart failure (CHF), liver disease, diabetes, renal disease, sepsis, and the Charlson Comorbidity Index (CCI). Details of organ support therapies, particularly mechanical ventilation (MV), were documented. Illness severity was quantified using standardized scoring systems, including the Oxford Acute Severity of Illness Score (OASIS), Simplified Acute Physiology Score (SAPS II), and the sequential organ failure assessment (SOFA) score.

Survival outcomes and ICU length of stay were extracted from the “demographic ICU stay detail” table in the MIMIC-IV database. For continuous variables with minimal missing data (<5%), mean or median imputation strategies were used to address the missing values.

### 2.4. Outcome and clinical definitions

The primary outcome of interest was the onset of AKI within the first 7 days of ICU admission. AKI was defined according to the kidney disease: improving global outcomes criteria. This definition includes an increase in SCr of 0.3 mg/dL or more above baseline within a 48-hour period, or a urine output of <0.5 mL/kg/h over a 6-hour duration.^[8]^

### 2.5. Sensitivity analysis

A sensitivity analysis was conducted to account for potential confounders in the study cohort. Patients with a preexisting diagnosis of hepatorenal syndrome prior to ICU admission were excluded specifically, as these patients are often treated with substantial intravenous albumin supplementation, which could skew the results and affect the reliability of the findings related to albumin levels.^[9]^

### 2.6. Statistical analysis

Patient characteristics were categorized according to the tercile distribution of the RAR. Continuous variables were expressed as either mean ± standard deviation or median with interquartile range, depending on the distribution. Categorical variables were reported as frequency and percentage. Comparative analyses were conducted using the Chi-square test for categorical variables, one-way ANOVA for normally distributed continuous variables, and the Kruskal–Wallis test for non-normally distributed continuous variables.

Univariate and multivariate logistic regression models to assess the association between RAR and the onset of AKI within 7 days of ICU admission among patients with AP were employed. The models were structured as follows: Model 1 was adjusted for age, race, and sex; Model 2 included further adjustments for sex, race, age, CHF, liver disease, renal disease, diabetes, sepsis, and the CCI; Model 3 built upon Models 1 and 2, with additional adjustments for mean blood pressure, glucose, hemoglobin, white blood cell (WBC) count, anion gap, creatinine, potassium, APTT, MV, and the SOFA score.

Curve fitting was applied to investigate the linear relationship between RAR and the probability of AKI onset within the first 7 days of ICU admission in patients with AP. Subgroup analyses were performed using stratified linear regression models and likelihood ratio tests to identify potential modifications and interactions. All statistical analyses were carried out using R software (version 4.2.1; R Foundation for Statistical Computing, http://www.R-project.org) and Free Statistics software (version 1.7, Beijing Free Clinical Medical Technology Co., Ltd, Beijing, China). A 2-sided *P*-value of <.05 was considered statistically significant.

## 3. Results

### 3.1. Patient baseline characteristics

A total of 599 patients were eligible based on the predefined inclusion criteria, as detailed in Figure 1. The baseline characteristics of these patients are detailed in Table 1. Patients were categorized into 3 groups based on their RAR values: low (2.61–4.27), medium (4.28–5.44), and high (5.45–14.61). The cohort had a mean age of 58.3 ± 17.8 years, consisting of 258 (43.1%) females and 341 (56.9%) males.

**Table 1:** Characteristics of the study patients.

Characteristics	RAR				*P*-value
	Total, n = 599	T1 (2.61–4.27), n = 200	T2 (4.27–5.44), n = 199	T3 (5.44–14.61), n = 200	
*Demographics*					
Sex, n					.006
Female	258 (43.1)	68 (34)	96 (48.2)	94 (47)	
Male	341 (56.9)	132 (66)	103 (51.8)	106 (53)	
Age	58.3 ± 17.8	57.3 ± 19.5	59.5 ± 17.0	57.9 ± 16.7	.435
Race					.963
White	372 (62.1)	123 (61.5)	125 (62.8)	124 (62)	
Other	227 (37.9)	77 (38.5)	74 (37.2)	76 (38)	
MBP (mm Hg)	83.1 ± 13.3	86.9 ± 14.0	82.9 ± 12.8	79.6 ± 12.0	<.001
Resp rate (beats/min)	21.0 ± 4.4	20.0 ± 4.2	21.3 ± 4.3	21.7 ± 4.6	<.001
Laboratory parameters					
Hemoglobin (g/dL)	10.6 ± 2.2	11.6 ± 2.0	10.7 ± 2.1	9.5 ± 2.0	<.001
WBC (10^9^/L)	16.1 ± 9.0	15.5 ± 8.4	15.3 ± 8.7	17.6 ± 9.7	.019
RDW (%)	15.0 ± 1.9	14.0 ± 1.2	15.0 ± 1.6	16.0 ± 2.1	<.001
Glucose (mmol/L)	8.2 ± 3.3	8.1 ± 3.1	7.9 ± 3.1	8.5 ± 3.5	.139
Serum albumin (g/dL)	3.0 ± 0.7	3.7 ± 0.4	3.0 ± 0.4	2.3 ± 0.4	<.001
RAR	5.3 ± 1.6	3.8 ± 0.4	4.9 ± 0.4	7.0 ± 1.4	<.001
Anion gap (mmol/L)	19.1 ± 6.9	20.0 ± 6.8	18.4 ± 6.3	19.1 ± 7.4	.064
Calcium (mmol/L)	7.5 ± 1.1	7.8 ± 1.0	7.6 ± 1.0	7.2 ± 1.1	<.001
Chloride (mmol/L)	101.5 ± 7.7	99.5 ± 7.4	102.4 ± 7.3	102.7 ± 7.8	<.001
Potassium (mmol/L)	3.7 ± 0.6	3.7 ± 0.5	3.7 ± 0.6	3.7 ± 0.6	.759
BUN (mg/dL)	23.0 (14.0, 42.0)	22.0 (13.8, 36.0)	19.0 (13.0, 38.5)	28.0 (16.0, 50.0)	.003
Creatinine (mEq/L)	1.2 (0.8, 2.2)	1.1 (0.8, 1.8)	1.1 (0.8, 2.0)	1.4 (0.9, 3.0)	.066
APTT (s)	41.2 ± 25.9	37.7 ± 24.6	38.6 ± 22.5	46.9 ± 29.1	<.001
Bilirubin total (mg/dL)	1.2 (0.7, 3.5)	1.2 (0.7, 3.1)	1.2 (0.6, 3.7)	1.4 (0.6, 4.4)	.675
*Comorbidities, n*					
CHF					.192
No	499 (83.3)	159 (79.5)	168 (84.4)	172 (86)	
Yes	100 (16.7)	41 (20.5)	31 (15.6)	28 (14)	
Liver disease					.007
No	409 (68.3)	152 (76)	134 (67.3)	123 (61.5)	
Yes	190 (31.7)	48 (24)	65 (32.7)	77 (38.5)	
Diabetes					.097
No	429 (71.6)	135 (67.5)	140 (70.4)	154 (77)	
Yes	170 (28.4)	65 (32.5)	59 (29.6)	46 (23)	
Renal disease					.518
No	514 (85.8)	167 (83.5)	173 (86.9)	174 (87)	
Yes	85 (14.2)	33 (16.5)	26 (13.1)	26 (13)	
Sepsis					<.001
No	201 (33.6)	92 (46)	62 (31.2)	47 (23.5)	
Yes	398 (66.4)	108 (54)	137 (68.8)	153 (76.5)	
MV					<.001
No	367 (61.3)	151 (75.5)	124 (62.3)	92 (46)	
Yes	232 (38.7)	49 (24.5)	75 (37.7)	108 (54)	
*Scoring systems*					
CCI	4.0 (3.0,6.0)	4.0 (2.0,6.0)	4.0 (3.0,6.0)	4.0 (3.0,6.0)	.1
SAPSII	37.3 ± 16.8	31.2 ± 15.0	36.8 ± 15.7	43.7 ± 17.4	<.001
OASIS	34.8 ± 10.4	30.4 ± 9.5	34.9 ± 10.1	39.0 ± 9.7	<.001
SOFA	5.0 (3.0, 8.0)	4.0 (2.0, 6.0)	5.0 (3.0, 8.0)	7.0 (4.0, 10.0)	<.001
*Outcome*					
AKI					<.001
No	231 (38.6)	113 (56.5)	79 (39.7)	39 (19.5)	
Yes	368 (61.4)	87 (43.5)	120 (60.3)	161 (80.5)	
28-day mortality					<.001
No	520 (86.8)	190 (95)	184 (92.5)	146 (73)	
Yes	79 (13.2)	10 (5)	15 (7.5)	54 (27)	

*P*-values were calculated using chi-square test, one-way ANOVA, and Kruskal–Wallis test.

APTT = activated partial thromboplastin time, BUN = blood urea nitrogen, CCI = Charlson Comorbidity Index, CHF = congestive heart failure, MB = mean blood pressure, MV = mechanical ventilation, OASIS = Oxford acute severity of illness score, RAR = red blood cell distribution width-to-albumin ratio, RDW = red cell distribution width, SAPS II = simplified acute physiology score, SOFA = sequential organ failure assessment, WBC = white blood cell.

Over the 7-day period following ICU admission, 368 (61.4%) patients developed AKI. Higher RAR values were associated with increased respiratory rates, WBC counts, RDW, chloride levels, and APTT. Conversely, patients in the higher RAR group exhibited lower blood pressure, body temperature, hemoglobin, serum albumin, and calcium levels. The high RAR group was more likely to have liver disease, sepsis, and a greater need for MV, as well as a higher 28-day mortality rate. Furthermore, these patients had significantly elevated scores on the OASIS, SAPS II, and SOFA, as detailed in Table 1.

### 3.2. Univariate logistic regression analysis of AKI incidence

RAR was identified as a significant predictor of AKI within 7 days following ICU admission, with higher RAR values associated with an increased risk of AKI (odds ratio = 1.7, 95% confidence interval [CI]: 1.47–1.96, *P* < .001), as detailed in Table 2. Univariate analysis also indicated significant associations between AKI and various factors, including race, mean blood pressure, respiratory rate, WBC count, glucose, albumin, RDW, electrolytes, renal function markers (blood urea nitrogen and creatinine), serum albumin, MV, comorbidities (CHF, renal disease, sepsis, CCI), and severity scores (OASIS, SAPS II, and SOFA), all with *P*-values < .05. Additional significant variables are detailed in Table S1, Supplemental Digital Content, https://links.lww.com/MD/Q171.

**Table 2:** Multivariate logistics regression of the association between different RAR levels and AKI occurs within 7 days.

Outcomes	Crude mode	*P*-value	Mode 1	*P* value	Mode 2	*P* value	Mode 3	*P* value
	OR (95% CI)		OR (95%CI)		OR (95%CI)		OR (95%CI)	
RAR	1.7 (1.47–1.96)	<.001	1.74 (1.5–2.01)	<.001	1.68 (1.44–1.96)	<.001	1.62 (1.34–1.96)	<.001
*Quintiles*								
T1 (2.61–4.27)	1 (Ref)		1 (Ref)		1 (Ref)		1 (Ref)	
T2 (4.28–5.44)	1.97 (1.32–2.94)	.001	2.08 (1.38–3.13)	<.001	1.96 (1.26–3.06)	.003	2.13 (1.3–3.5)	.003
T3 (5.45–14.61)	5.36 (3.43–8.39)	<.001	5.86 (3.7–9.29)	<.001	5.42 (3.29–8.92)	<.001	4.64 (2.58–8.33)	<.001
*P* for trend		<.001		<.001		<.001		<.001

Logistics proportional hazard regression models were used to calculate odds ratio (OR) with 95% confidence intervals; crude model was adjusted for none; Model 1 was adjusted for age race and gender; Model 2 was adjusted for Model 1 + (CHF + liver disease + renal disease + diabetes + sepsis + CCI); Model 3 was adjusted for Model 1 + Model 2 + (MBP + glucose + hemoglobin + WBC + anion gap + creatinine + potassium + APTT + MV + SOFA).

APTT = activated partial thromboplastin time, CI = confidence interval, CCI = Charlson Comorbidity Index, CHF = congestive heart failure, MBP = mean blood pressure, MV = mechanical ventilation, RAR = red blood cell distribution width-to-albumin ratio, SOFA = sequential organ failure assessment, WBC = white blood cell.

### 3.3. Multivariate logistic regression analysis of RAR and AKI

In the multivariate analysis, after adjustments, RAR remained significantly associated with the occurrence of AKI, as detailed in Table 2. The risk of AKI increased by 62% with each unit increase in RAR (*P* < .001). Stratified analysis revealed that the incidence of AKI in the highest RAR subgroup (T3) was 3.64 times higher than that in the lowest RAR subgroup (T1) (*P* < .001).

### 3.4. Restricted cubic spline regression model

**Figure 2.**
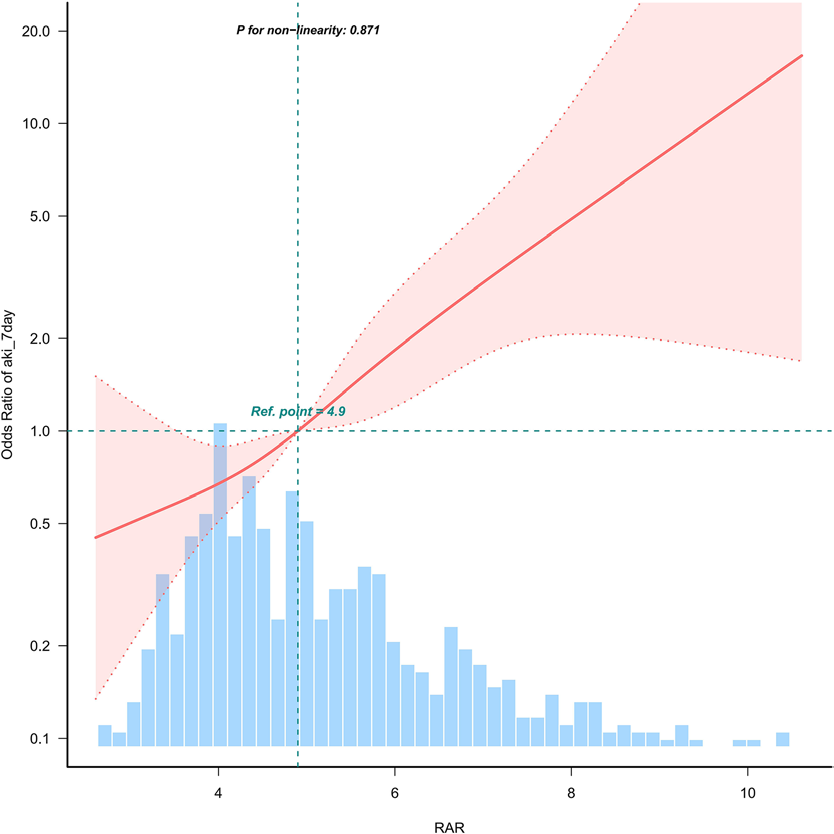
Association between RAR and early onset of AKI in patients with AP. RAR was analyzed as a continuous variable, with adjustments made for demographic, comorbidity, and clinical factors, including age, race, sex, CHF, Liver disease, renal disease, diabetes, sepsis, CCI, MBP, glucose, hemoglobin, WBC, anion gap, creatinine, potassium, APTT, MV, SOFA score. AP = acute pancreatitis, APTT = activated partial thromboplastin time, CCI = Charlson Comorbidity Index, CHF = congestive heart failure, MBP = mean blood pressure, MV = mechanical ventilation, RAR = red blood cell distribution width-to-albumin ratio, SOFA = sequential organ failure assessment, WBC = white blood cell.

The risk of AKI increased linearly with rising RAR levels, as detailed in Figure 2.

### 3.5. Subgroup analyses by adjusted potential effect confounders

**Figure 3.**
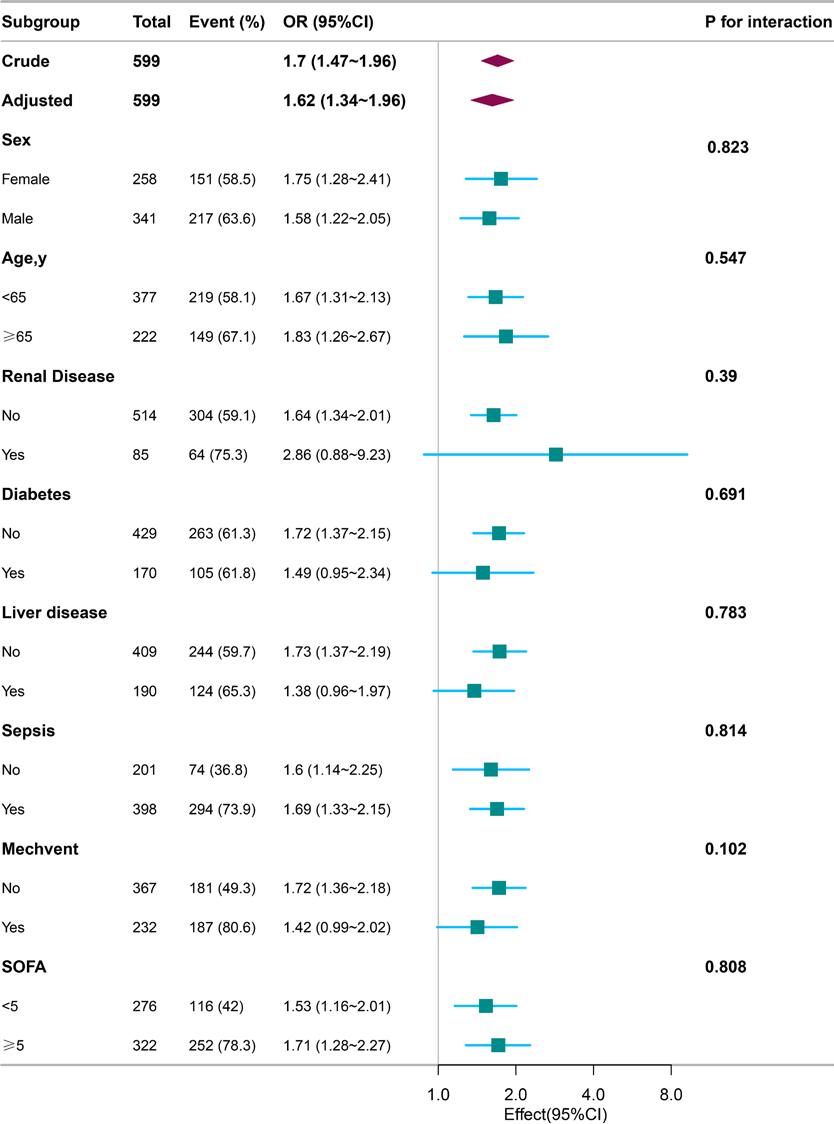
Subgroup analyses of RAR in patients with AP. Subgroup analyses evaluating the association between RAR and AKI incidence in different demographic and clinical conditions. Adjustments were made for demographic, comorbidity, and clinical factors, including age, race, sex, CHF, liver disease, renal disease, diabetes, sepsis, CCI, MBP, glucose, hemoglobin, WBC, anion gap, creatinine, potassium, APTT, MV, and SOFA score. AP = acute pancreatitis, APTT = activated partial thromboplastin time, CCI = Charlson Comorbidity Index, CHF = congestive heart failure, MBP = mean blood pressure, MV = mechanical ventilation, RAR = red blood cell distribution width-to-albumin ratio, SOFA = sequential organ failure assessment, WBC = white blood cell.

Subgroup analyses were performed to evaluate the relationship between RAR and the risk of AKI across diverse patient demographics and clinical conditions, including sex, age, renal disease, liver disease, diabetes, sepsis, SOFA scores, and the use of MV. The findings demonstrated consistent results, underscoring robustness across all subgroups (*P* > .05), as detailed in Figure 3.

### 3.6. Sensitivity analysis

In the sensitivity analyses, 19 patients with a pre-ICU diagnosis of hepatorenal syndrome were excluded. The association between RAR and AKI remained consistent, as detailed in Table S2, Supplemental Digital Content, https://links.lww.com/MD/Q171.

## 4. Discussion

This study found a significant correlation between RAR and the risk of AKI in patients with AP, as demonstrated in the adjusted model. Sensitivity analyses, excluding patients diagnosed with hepatorenal syndrome prior to ICU admission, confirmed that the association between RAR and AKI risk persisted. Subgroup analyses further validated the consistency of this correlation across various factors, including sex, age groups, underlying conditions, SOFA scores, and the use of MV, underscoring the robustness of the findings.

Previous studies have demonstrated that an elevated RAR is associated with adverse outcomes in inflammatory diseases. Meng Hao cohort study reported that higher RAR levels were linked to increased all-cause and specific-cause mortality.^[3]^ Similarly, Ni et al identified RAR as a predictor of mortality in patients with heart failure, while Hong et al highlighted its utility in identifying high-risk patients with diabetic foot ulcers.^[10,11]^ Despite these findings, limited research has explored the relationship between RAR and AKI occurrence. One survey confirmed an association between elevated RAR and a higher risk of AKI in sepsis (relative risk: 1.09, 95% CI: 1.02–1.16, *P* = .013).^[12]^ Distinct from the aforementioned populations, this study established a significant positive correlation between RAR levels at ICU admission and the risk of AKI within 1 week in patients with AP (relative risk: 1.62, 95% CI: 1.34–1.96, *P* < .001).

The mechanism linking elevated RAR in patients with AP to an increased risk of AKI remains unclear. This association may stem from the combined effects of increased RDW and decreased albumin during systemic inflammation. Potential pathophysiological mechanisms contributing to AKI in AP involve the release of activated enzymes and proteases, which increase vascular permeability, fluid extravasation, hypovolemia, elevated abdominal pressure, inflammatory cascades, renal vasoconstriction, intravascular coagulation dysfunction, and the release of nephrotoxic substances. These factors collectively mediate AKI development.^[2]^

During systemic inflammation, reduced erythropoietin levels suppress erythrocyte production while increasing erythrocyte fragmentation, leading to elevated RDW and greater red blood cell heterogeneity.^[13]^ Cai et al identified elevated RDW at admission as a predictor of AKI incidence and 28-day mortality in patients with acute respiratory distress syndrome.^[14]^ Similarly, a prospective observational study in a cardiac care unit reported a significantly higher incidence of AKI among patients with elevated RDW levels (≥14.0%).^[15]^

Serum albumin plays a critical role in renal protection through several mechanisms: it maintains renal perfusion, preserves the structural and functional integrity of proximal renal tubules, binds endogenous toxins and nephrotoxic drugs, prevents oxidative damage, and delivers protective lysophosphatidic acid.^[16]^ A large-scale study involving 2461 Hungarian patients with pancreatitis demonstrated that hypoalbuminemia dose-dependently increased the risks of severe pancreatitis, mortality, local complications, and organ failure, while also prolonging hospital stays.^[17]^ Inflammatory states suppress albumin synthesis, increase albumin consumption, and cause vascular leakage, reducing serum albumin levels. Monitoring RDW and albumin levels through the RDW/ALB ratio provides a stable and integrated measurement, making it a potentially more reliable predictor of AKI compared to RDW or albumin alone.

The evaluation of factors associated with AKI in patients with AP is crucial for improving prognosis prediction. Traditional markers such as SCr levels and urine output are widely used but have notable limitations. SCr typically increases only after renal damage has occurred, reducing their effectiveness for early AKI detection. While reduced urine output may provide an earlier indicator of AKI, the process of monitoring urine volume is time-consuming, labor-intensive, and prone to challenges in maintaining data accuracy and completeness. Recent studies have highlighted biomarkers such as neutrophil gelatinase-associated lipocalin and kidney injury molecule-1 as potential early diagnostic tools for AKI.^[18,19]^ However, their application in routine clinical practice is constrained by issues such as cost, availability, and the need for specialized assays. This underscores a pressing need for a simple, cost-effective, and reliable biomarker capable of early AKI prediction. The RAR addresses these needs effectively. As a parameter derived from routine laboratory tests, it is readily accessible in most clinical settings. The simplicity, low cost, and ease of obtaining RAR, combined with its demonstrated predictive value make it a strong candidate for early AKI risk assessment in patients with AP.

The study’s primary strength lies in its relatively large patient cohort, which allowed for detailed stratification and subgroup analysis of the association between RAR and the risk of AKI in patients with pancreatitis. However, several limitations must be acknowledged. First, as a retrospective cohort study, the inherent nature of retrospective data collection introduces potential biases. Second, the study was based on a subset of the population from the MIMIC-IV database, which may limit the generalizability of the results. Third, as an observational study, the possibility of residual confounding cannot be excluded. Future research should focus on conducting large-scale, multicenter prospective studies to validate the observed association between RAR and the risk of AKI in patients with AP.

## 5. Conclusions

This study provides novel evidence linking elevated RAR levels with an increased incidence of AKI in patients with AP upon ICU admission. The simplicity and clinical applicability of RAR highlight its potential as a tool for early risk stratification and prevention of AKI in this patient population. However, further validation through large-scale, prospective, and multicenter studies is warranted to confirm these preliminary insights.

## Author contributions

**Conceptualization:** Jian-Tao Zheng.

**Data curation:** Si-Ming Lin, Jian-Tao Zheng.

**Formal analysis:** Chan-Juan Zhou, Jian-Tao Zheng.

**Funding acquisition:** Chan-Juan Zhou, Jian-Tao Zheng.

**Software:** Chan-Juan Zhou, Si-Ming Lin.

**Writing – original draft:** Chan-Juan Zhou.

**Writing – review & editing:** Chan-Juan Zhou, Si-Ming Lin, Jian-Tao Zheng.

## Supplementary Material
