# Supplementary material for: Association between red cell distribution width-to-albumin ratio and acute kidney injury in acute pancreatitis: A retrospective cohort study from the MIMIC-IV database
Source: Medicine (Baltimore). 2025 Oct 3;104(40):e44810. doi: 10.1097/MD.0000000000044810 (PMC12499778; doi:10.1097/MD.0000000000044810)
Supplement: Supplementary file 1 [file medi-104-e44810-s001.pdf]

Supplemental Digital Content 1. The results of univariate analysis

Variable	OR (95%CI)	P value
Sex	1.3 (0.92~1.82)	0.136
Age	1.01 (1~1.02)	0.077
Race	0.7 (0.5~0.99)	0.044
MBP (mmHg)	0.97 (0.96~0.98)	<0.001
Resp rate (beats/min)	1.08 (1.04~1.13)	<0.001
Glucose (mmol/L)	1.09 (1.03~1.16)	0.002
Hemoglobin	0.93 (0.86~1.01)	0.08
WBC (10^9/L)	1.05 (1.02~1.07)	<0.001
Serum Albumin (g/dL)	0.4 (0.3~0.52)	<0.001
Calcium (mmol/L)	0.63 (0.53~0.76)	<0.001
RDW	1.16 (1.05~1.28)	0.004
RAR	1.68 (1.44~1.95)	<0.001
Anion gap (mmol/L)	1.02 (0.99~1.04)	0.18
Potassium (mmol/L)	1.6 (1.16~2.2)	0.004
BUN (mg/dl)	1.02 (1.01~1.03)	<0.001
Creatinine (mEq/L)	1.48 (1.28~1.71)	<0.001
Bilirubin total (mg/dL)	1.12 (1.05~1.19)	<0.001
APTT (s)	1.01 (1~1.02)	0.004
CHF	1.93 (1.19~3.14)	0.008
Liver disease	1.24 (0.86~1.8)	0.248

Diabetes	1.03 (0.71~1.49)	0.879
Renal disease	2.26 (1.32~3.86)	0.003
Sepsis	4.67 (3.23~6.76)	<0.001
SAPSII	1.07 (1.06~1.09)	<0.001
OASIS	1.13 (1.1~1.16)	<0.001
CCI	1.09 (1.03~1.17)	0.005
SOFA	1.32 (1.25~1.41)	<0.001
MV	4.11 (2.78~6.09)	<0.001

RAR (red blood cell distribution width-to-albumin ratio), MBP (mean blood pressure), WBC (white blood cell), RDW (red cell distribution width), BUN (blood urea,) APTT (activated partial thromboplastin time), CHF (congestive heart failure), MV (mechanical ventilation), CCI (Charlson comorbidity index), OASIS (Oxford acute severity of illness score), SAPS II (simplified acute physiology score), SOFA (sequential organ failure assessment).

P-values were calculated using chi-square test, one-way ANOVA, and Kruskal–Wallis test.

Supplemental Digital Content 2. Multivariate Logistics regression of the association between different RAR levels and AKI occurs within 7 days

Outcomes	n. total	n. event (%)	crude. OR (95%CI)	P value	adj. OR (95%CI)	P value
RAR	580	350 (60.3)	1.69 (1.46~1.96)	<0.001	1.64 (1.35~1.99)	<0.001
T1(2.61~4.36)	197	84 (42.6)	1(Ref)		1(Ref)	
T2(4.37~5.64)	194	115 (59.3)	1.96 (1.31~2.93)	0.001	2.1 (1.28~3.45)	0.003
T3(5.65~14.62)	189	151 (79.9)	5.35 (3.39~8.42)	<0.001	4.85 (2.68~8.8)	<0.001
P for trend	580	350 (60.3)		<0.001		<0.001

After excluding patients with liver and kidney syndrome, Logistics proportional hazard regression models were used to calculate Odds ratio (OR) with 95% confidence intervals. Odds ratio were adjusted for age, race, gender, CHF, Liver disease, renal disease, diabetes, sepsis, CCI, MBP, glucose, hemoglobin, WBC, anion gap, creatinine, potassium, APTT, MV, SOFA.

RAR (red blood cell distribution width-to-albumin ratio), CHF (congestive heart failure), CCI (Charlson comorbidity index), MBP (mean blood pressure), WBC (white blood cell), APTT (activated partial thromboplastin time), MV (mechanical ventilation), SOFA (sequential organ failure assessment).
